# Prognostic Role and Diagnostic Power of Seven Indicators in COVID-19 Patients

**DOI:** 10.3389/fmed.2021.733274

**Published:** 2021-10-27

**Authors:** Lili Ding, Wanwan Zhang, Fengling Zhang, Chaoqun Huang, Ming Yang, Zhouping Tang, Yongwu Li, Jun Mi, Weimin Zhong

**Affiliations:** ^1^The Fifth Hospital of Xiamen, Xiamen, China; ^2^Affiliated Union Hospital Pingtan Branch, Fujian Medical University, Fuzhou, China; ^3^Tongji Hospital, Tongji Medical College, Huazhong University of Science and Technology, Wuhan, China

**Keywords:** COVID-19, indicators, diagnostic biomarker, prognostic model, survival analysis

## Abstract

The prognostic role and diagnostic ability of coronavirus disease 2019 (COVID-19) disease indicators are not elucidated, thus, the current study aimed to investigate the prognostic role and diagnostic ability of several COVID-19 disease indicators including the levels of oxygen saturation, leukocytes, lymphocytes, albumin, C-reactive protein (CRP), interleukin-6 (IL-6), and D-dimer in patients with COVID-19. The levels of oxygen saturation, lymphocytes, and albumin were significantly higher in the common and severe clinical type patients compared with those in critical type patients. However, levels of leukocytes, CRP, IL-6, and D-dimer were significantly lower in the common and severe type patients compared with those in critical type patients (*P* < 0.001). Moreover, the current study demonstrated that the seven indicators have good diagnostic and prognostic powers in patients with COVID-19. Furthermore, a two-indicator (CRP and D-dimer) prognostic signature in training and testing datasets was constructed and validated to better understand the prognostic role of the indicators in COVID-19 patients. The patients were classified into high-risk and low-risk groups based on the median-risk scores. The findings of the Kaplan–Meier curve analysis indicated a significant divergence between the high-risk and low-risk groups. The findings of the receiver operating curve (ROC) analysis indicated the good performance of the signature in the prognosis prediction of COVID-19. In addition, a nomogram was constructed to assist clinicians in developing clinical decision-making for COVID-19 patients. In conclusion, the findings of the current study demonstrated that the seven indicators are potential diagnostic markers for COVID-19 and a two-indicator prognostic signature identification may improve clinical management for COVID-19 patients.

## Introduction

The coronavirus disease-2019 (COVID-19) outbreak has rapidly spread out globally since the confirmation of the first case in Wuhan city (China) in December 2019 (1). The prevalence and mortality rates of COVID-19 have exceeded those of the Middle East Respiratory Syndrome Coronavirus (MERS-CoV) and Severe Acute Respiratory Syndrome (SARS) (2). The WHO declared COVID-19 as a significant threat to international health and a global pandemic. The real-time statistics from Johns Hopkins University reported more than 17.53 million confirmed COVID-19 cases and nearly 680,000 COVID-19 deaths worldwide as of August 1, 2020. Previous studies reported that most SARS-CoV-2 patients have a better prognosis and could gradually recover after 2 weeks. In addition, a small number of patients developed severe pneumonia (3–5), acute respiratory distress syndrome (ARDS), or Multiple Organ Dysfunction Syndrome, which lead to death. The levels of interleukin-6 (IL-6), D-dimer, and lymphocytes could help clinicians to identify COVID-19 patients with poor prognoses early and identify high-risk patients (6–8).

The latest COVID-19 studies focus on patients with severe and critical illnesses. This is because this stage of disease progression may lead to rapid deterioration, which can result in inflammatory storms, respiratory distress, multiple organ failure, and death (9). Currently, limited medical approaches and treatments are available for severe COVID-19. Therefore, it is important to explore effective prognostic predictors for timely intervention. Previous studies have reported that high expression levels of IL-6 and interleukin-10 (IL-10) can predict deterioration in patients with COVID-19 (10). In addition, previous studies have reported that D-dimer elevation, leukopenia, and thrombocytopenia are independent risk factors for severe COVID-19 (11). Furthermore, a D-dimer associated with pneumonia progression (12) and inpatient mortality (3, 6, 13–15) has been identified. A cohort study in Shanghai demonstrated that some factors including age (>64 years old), procalcitonin, D-dimer, C-reactive protein (CRP), lactate dehydrogenase, lymphocytes, neutrophils, CD4%, and CD4/CD8 ratio were considered as potential markers of disease progression (9, 16). Therefore, it is imperative to explore the early indicators of the progression of the disease to provide basic prediction and disease management for improving prognosis.

In the current study, a total of 104 COVID-19 patients from the Tongji Hospital Affiliated with Tongji medical college HUST (Wuhan, China) were included as study participants. The diagnostic abilities and levels of indicators among the common, severe, and critical type COVID-19 patients were evaluated using bioinformatics analysis. In addition, a survival analysis was undertaken and a two-indicator prognostic signature was developed to demonstrate the association between indicators and prognosis in COVID-19 patients.

## Materials and Methods

### Recruitment of Study Participants

A total of 104 adult inpatients (≥18 years old) with laboratory-confirmed COVID-19 status from Tongji Hospital affiliated with Tongji medical college HUST (Wuhan, China) were recruited in the current study. The patients had either been discharged or died between February 10 and March 28, 2020, after the exclusion of their incomplete clinical information. The patients were further classified as common (*N* = 50), severe type (*N* = 29), and critical types (*N* = 25) based on the following detailed criteria:

(1) Common type: Patients having cough, fever, and other symptoms including imaging finding of pneumonia; (2) Severe type: Patients with respiratory distress and respiratory rate ≥30 per min; patients whose oxygen saturation of indoor air at rest is ≤ 93%; and patients whose partial pressure of oxygen in arterial blood/fraction of inspired oxygen is ≤ 300 mmHg; and (3) Critical type: Patients with respiratory failure and required mechanical ventilation; patients with shock; and patients with other organ dysfunctions, requiring monitoring and treatment in the intensive care unit.

### Construction of the Prognostic Model

A univariate Cox regression analysis was conducted on the seven indicators in the study patients using the “survival” R package. A log-rank test was also done to screen the significant candidate indicators. The overall survival-related indicators were further screened based on the least absolute shrinkage and selection operator (LASSO) algorithm using the “glmnet” R package. In addition, the risk formula was constructed based on the level and coefficient of the indicators:

Risk score = Σ(Coef_indicators_ × Exp_indicators_), where the Coef_indicators_ represent the lasso coefficient of each indicator, Exp_indicators_ represent the level of each indicator. The risk score for each patient was computed based on the risk formula. A Kaplan–Meier (K–M) curve analysis was conducted to estimate the survival difference between the high-risk and low-risk groups. A receiver operating characteristic (ROC) analysis was done to evaluate the accuracy of the risk model and seven indicators.

### Statistical Analysis

The clinical data were classified into continuous and categorical data. For the continuous data, the differences were computed using a Kruskal–Wallis test. The categorical data were computed using a Chi-square test or Fisher exact test. The data were considered statistically significant if *P* < 0.05. Moreover, the survival differences of each indicator were estimated using the “survminer” R package based on the best separation level. A nomogram was constructed based on the seven indicators using the “rms” package. All analyses were conducted in the R environment.

## Results

### The Landscape of the Seven Indicators

**Figure 1:**
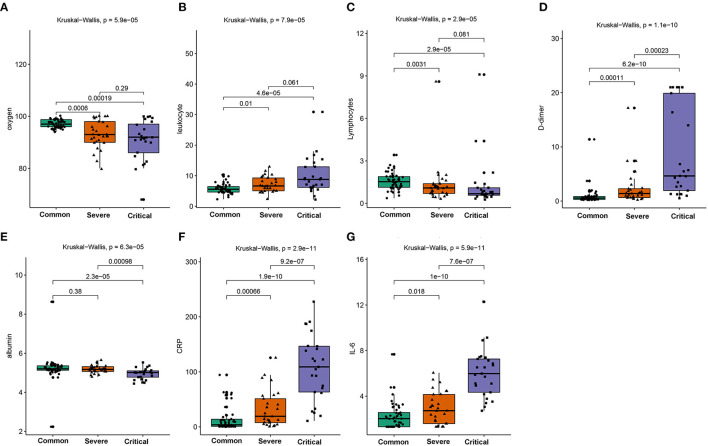
Comparison of the oxygen saturation **(A)**, leukocytes **(B)**, lymphocytes **(C)**, D-dimer **(D)**, albumin **(E)**, CRP **(F)**, and IL-6 **(G)** in the common, severe, and critical types of COVID-19 patients.

**Figure 2:**
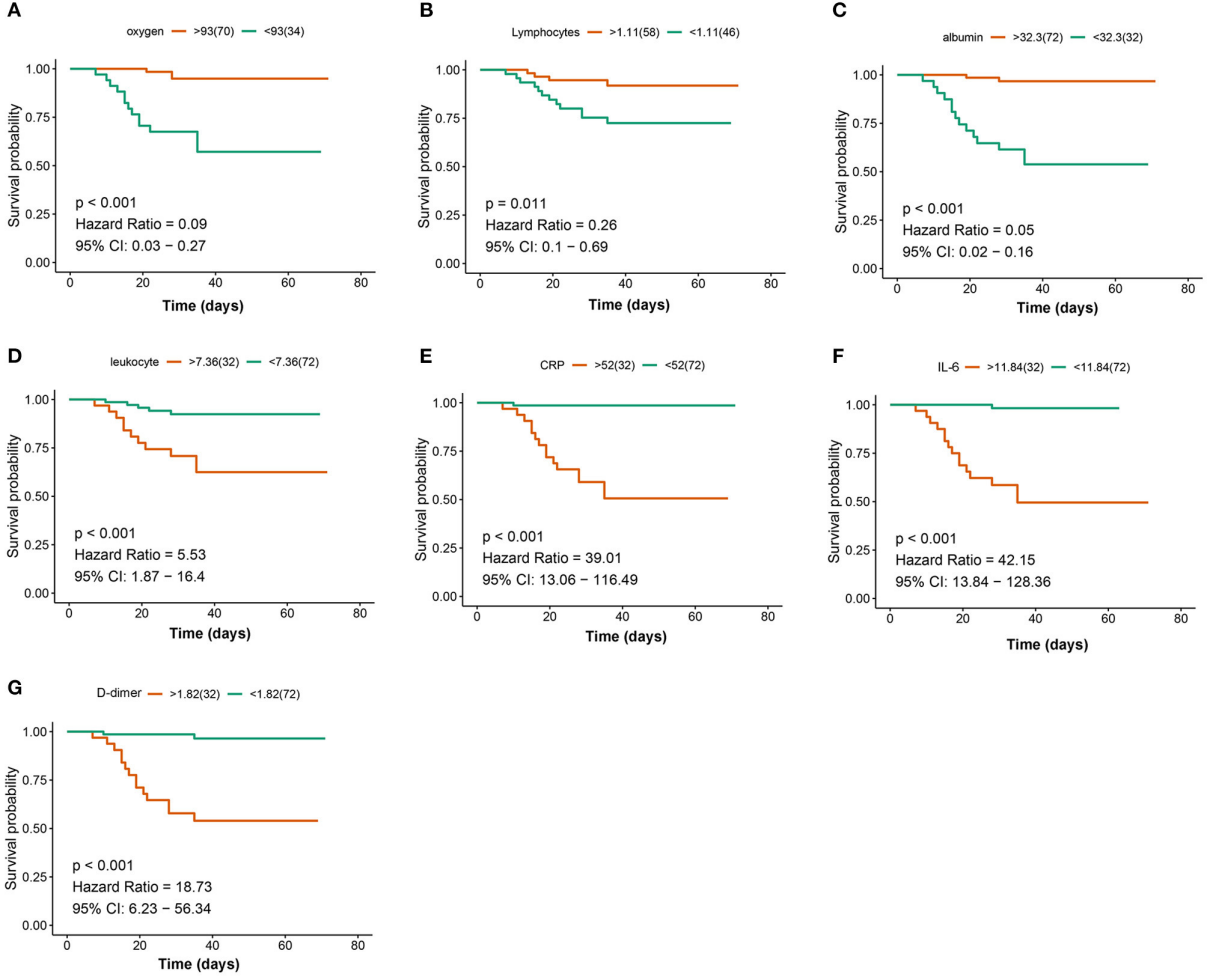
Kaplan–Meier (K–M) curves analysis for the seven indicators: oxygen **(A)**, lymphocytes **(B)**, albumin **(C)**, leukocyte **(D)**, C-reactive protein (CRP) **(E)**, interleukin-6 (IL-6) **(F)**, and D-dimer **(G)**.

**Figure 3:**
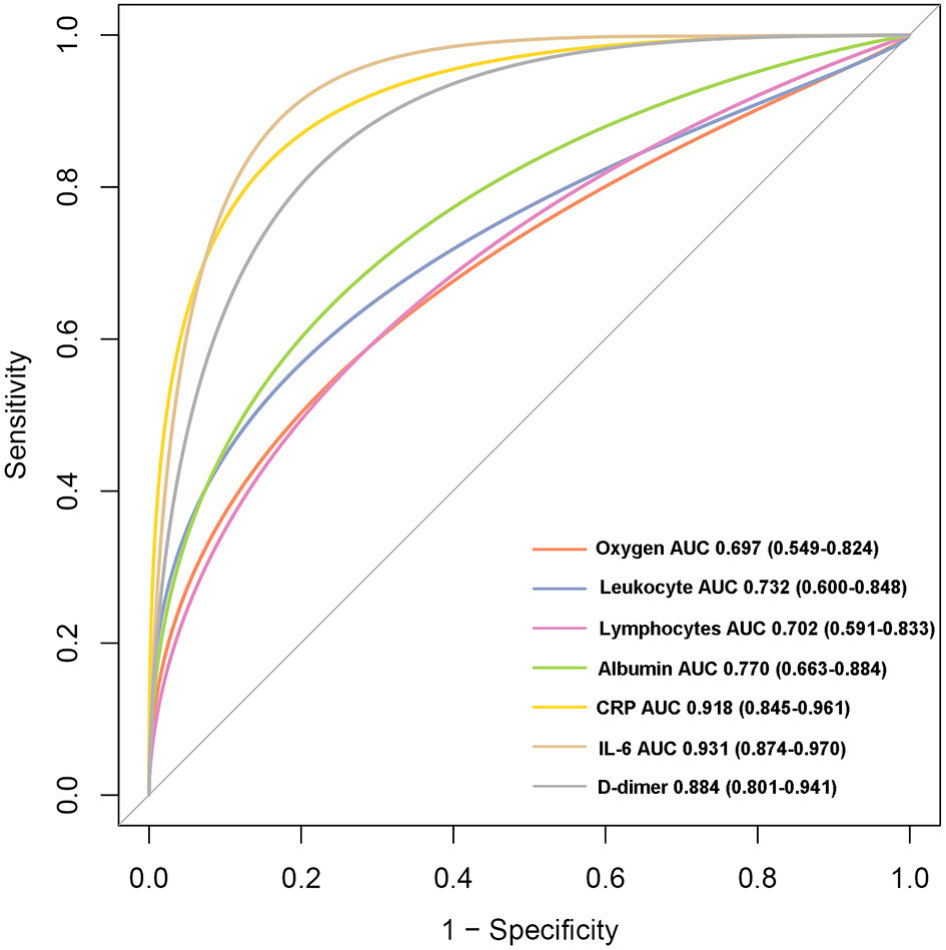
The receiver operating curve (ROC) analysis for the seven indicators between critical and non-critical types.

A total of 104 COVID-19 patients (alive: *N* = 88; dead: *N* = 16) were included in the current study after excluding patients based on the predefined exclusion criteria. According to the clinical type of COVID-19, the patients were classified into the common (*N* = 50), severe (*N* = 29), and critical types (*N* = 25) (Table 1). The expression levels of the seven indicators in the common, severe, and critical clinical types were compared. The findings of the current study established that the levels of oxygen, lymphocytes, and albumin in patients were significantly higher in the common, compared with those in the severe and critical clinical types (Figures 1A–G). In converse, the levels of leukocytes, CRP, IL-6, and D-dimer were significantly lower in the common, compared with those in the severe and critical clinical types (Kruskal–Wallis *P* < 0.001). A K–M curve analysis was conducted to further evaluate the prognostic value of the seven indicators. The current study findings demonstrated that oxygen saturation, lymphocytes, and albumin levels were protective factors (HR < 1), where their high levels prolonged survival time and improved the survival probability. However, the leukocyte, CRP, IL-6, and D-dimer levels were risk factors, whose high levels correspondingly led to poor survival outcomes (Figure 2). The ROC analysis revealed that the seven indicators have a good performance in the prognosis of COVID-19 patients (Supplementary Figure 1). In addition, the current study evaluated the diagnostic value of the seven indicators in non-critical and critical cases using ROC analysis. The seven indicators exhibited good diagnostic performance (Figure 3). Notably, IL-6 had the highest area under the curve (AUC) value (AUC = 0.931), whereas CRP and D2 were ranked second (AUC = 0.918) and third (AUC = 0.884), respectively. These results show that the seven indicators are potential diagnostic markers of COVID-19 in patients.

**Table 1:** The clinical information of the 104 COVID-19 patients.

**Items**	**Sub-items**	**Case distribution (104)**	**Common (50)**	**Severe (29)**	**Critical (25)**	***df***	***P***
Age (years)	Median	62.82 (14.77)	58.10 (14.82)	65.86 (14.94)	68.72 (11.54)	5.629	0.005
	≥60	69 (66.35%)	27 (39.13%)	21 (30.43%)	21 (30.43%)	7.381	0.025
Gender	Male	53 (50.96%)	23 (46.00%)	13 (44.83%)	17 (68.00%)	3.833	0.147
	Female	51 (49.04%)	27 (54.00%)	16 (55.17%)	8 (32.00%)		
Comorbidity		45 (49.21%)	8 (16%)	16 (55.17%)	21 (84.00%)	33.717	<0.0001
	High blood pressure	28 (26.92%)	2 (4%)	9 (31.03%)	17(68.00%)	35.043	<0.0001
	Diabetes mellitus	16 (15.38%)	4 (8%)	7 (24.14%)	5 (20.00%)	4.211	0.122
	Chronic obstructive pulmonary disease	5 (4.81%)	2 (4.0%)	1 (3.45%)	2 (8.80%)	0.689	0.745
	Coronary heart disease	8 (7.69%)	1 (2.00%)	3 (10.34%)	4 (16.00%)	4.999	0.082
	Cerebrovascular diseases	5 (4.81%)	1 (2.00%)	1 (3.45%)	3 (12.00%)	3.804	0.149
Oxygen saturation (%)		94.42 ± 5.50	97.10 ± 1.594	93.03 ± 5.302	90.68 ± 7.734	16.450	<0.0001
Primary leukocyte count		7.20 ± 3.81	5.75 ± 1.56	7.19 ± 2.50	10.10 ± 6.07	13.481	<0.0001
Primary lymphocyte count		1.29 ± 0.70	1.56 ± 0.61	1.09 ± 0.50	0.95 ± 0.84	9.141	<0.0001
Primary albumin		34.96 ± 5.57	36.72 ± 4.22	35.77 ± 5.14	30.48 ± 6.15	13.519	<0.0001
Primary C-reactive protein		41.71 ± 53.00	13.71 ± 21.02	33.04 ± 32.78	107.78 ± 60.23	54.76	<0.0001
Primary IL-6		87.85 ± 516.25	9.65 ± 31.07	12.02 ± 14.99	304.16 ± 987.83	3.112	0.049
Primary D-dimer		3.28 ± 5.63	0.91 ± 1.63	2.42 ± 3.42	9.01 ± 8.38	26.448	<0.0001

### Construction of Prognosis Signature

**Figure 4:**
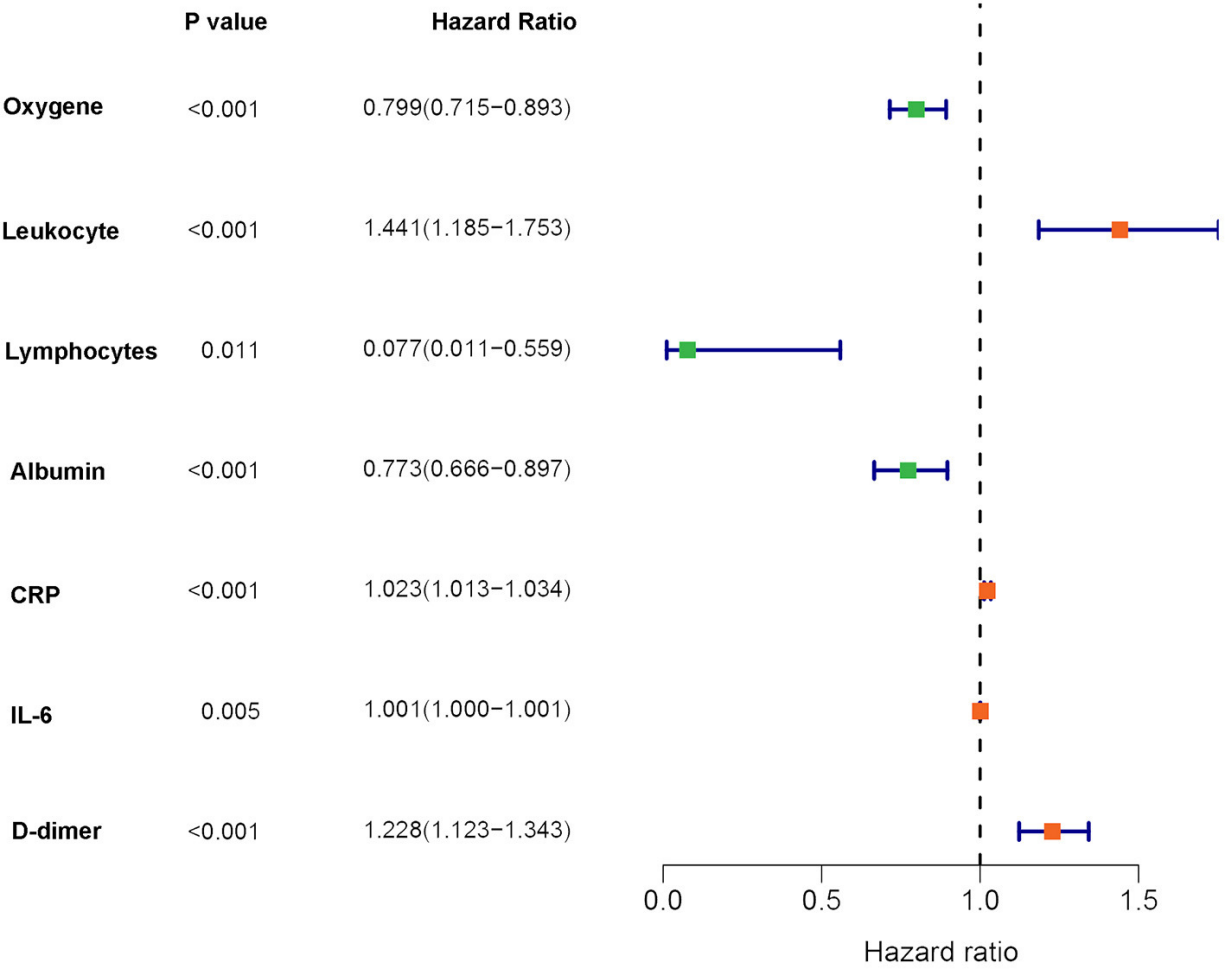
Forest plot for the univariate Cox regression analysis results in the training dataset.

**Figure 5:**
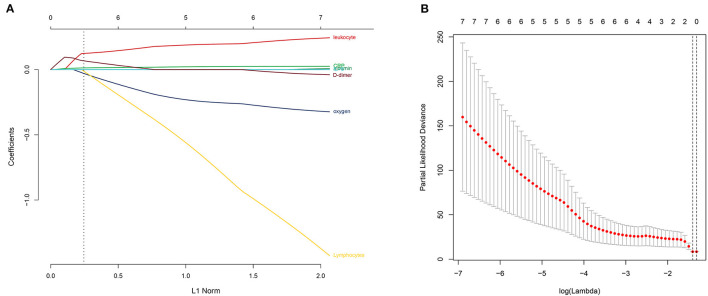
Selection of the prognostic signature using the least absolute shrinkage and selection operator (LASSO) analysis in the training dataset. **(A)** The LASSO coefficient spectrum of the indicators of COVID-19. **(B)** Selecting the best parameters for COVID-19 in the LASSO model (λ).

To better understand the prognostic roles of the seven indicators in COVID-19 patients, patients were equally classified into a training dataset (*N* = 54) and a testing dataset (*N* = 54). The levels of the seven indicators in the patients with COVID-19 were evaluated using a univariate Cox regression analysis in the training dataset. The findings showed that the seven indicators were significantly associated with the overall survival of COVID-19 patients (Figure 4). In addition, the seven indicators were subjected to a LASSO regression analysis. Two indicators (CRP and D-dimer) were then selected based on the minimum criteria to construct a risk signature using the coefficients derived from the LASSO algorithm (Figure 5). The risk scores for each patient in the training and testing datasets were computed based on the risk formula:

Risk score = 0.0011 ^*^ CRP level + 0.0345 ^*^ D-dimer level

**Figure 6:**
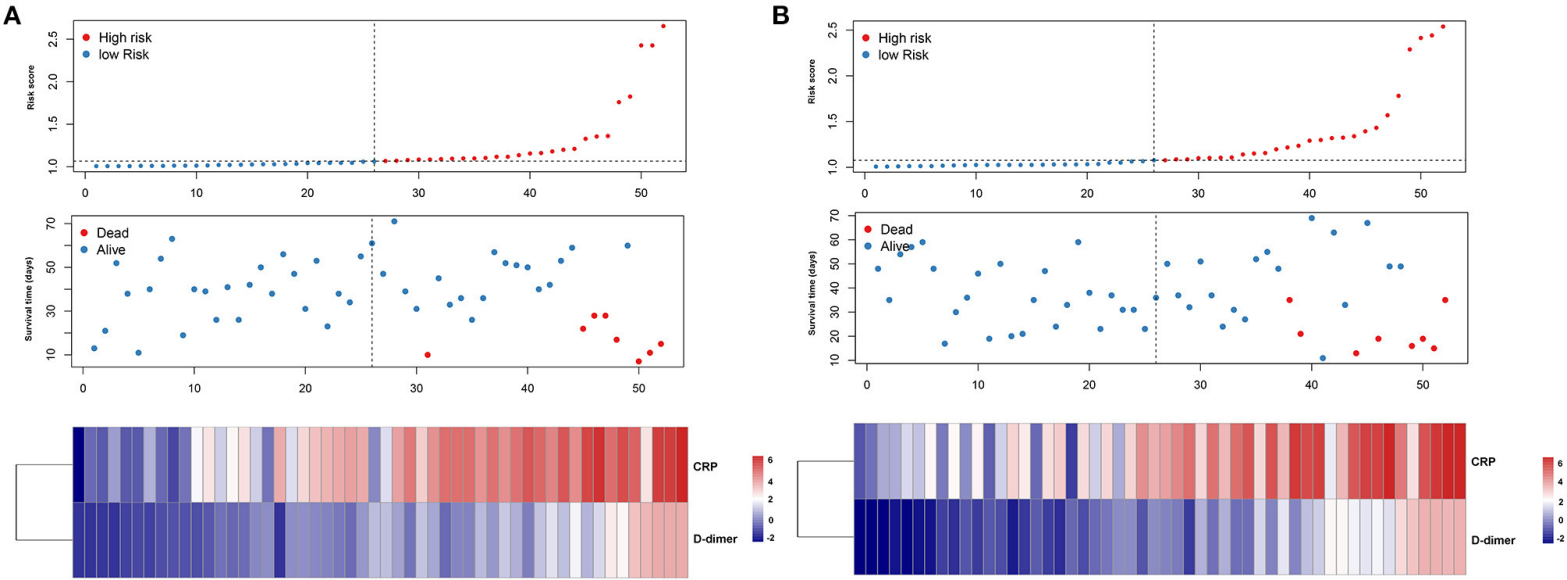
Characteristics of prognostic indicator signature in the training dataset **(A)** and testing dataset **(B)**, respectively. The upper panel showed the risk score distribution of the prognostic signature, the middle panel represent the patients distribution, and the lower panel represent the level of the two prognostic indicators.

**Figure 7:**
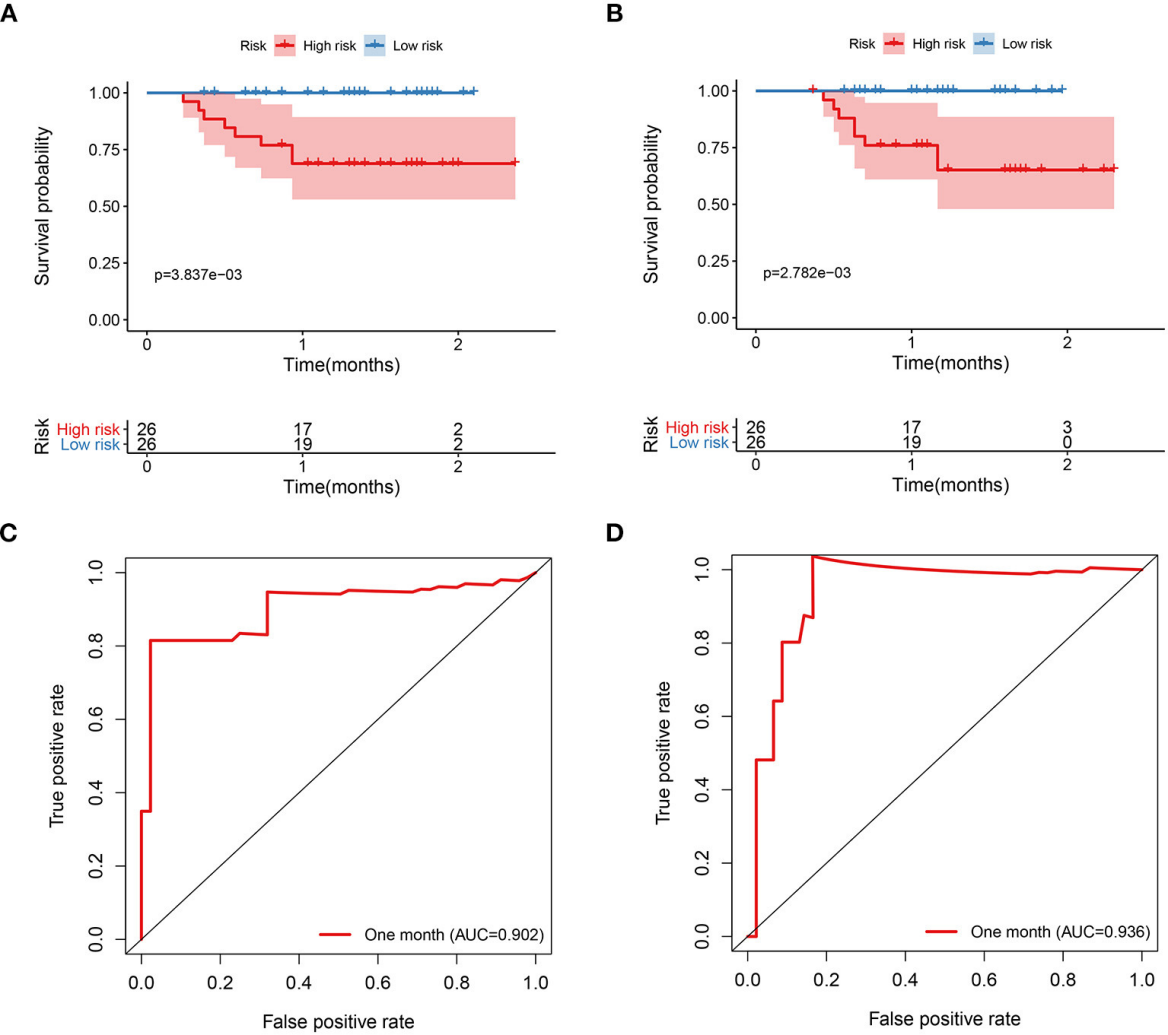
The prognostic signature is associated with the survival of patients with COVID-19. **(A)** The K–M curves analysis of COVID-19 patients that stratified patients by median risk score in the training dataset. **(B)** The K–M curves analysis of the COVID-19 patients that stratified patients by median risk score in the testing dataset. **(C)** The ROC analysis of the prognostic signature in the training dataset. **(D)** The ROC analysis of the prognostic signature in the testing dataset.

The patients were further categorized based on the risk scores into the high-risk and low-risk groups in the training and testing datasets, respectively. The findings of the current study established that most of the deceased cases corresponded with the high-risk group, whereas the alive cases were associated with the low-risk group (Figure 6). Moreover, patients in the high-risk group had high CRP and D-dimer levels in the training and testing datasets. The findings of the K–M curve analysis indicated a significant divergence in the overall survival outcomes between the risk groups in the training and testing datasets (*P* < 0.01) (Figures 7A,B). In addition, the findings of the ROC analysis showed that the risk signature accurately predicts the survival rate of patients in 1 month in the training and testing datasets (AUC > 0.90) (Figures 7C,D).

### Construction and Validation of the Nomogram

**Figure 8:**
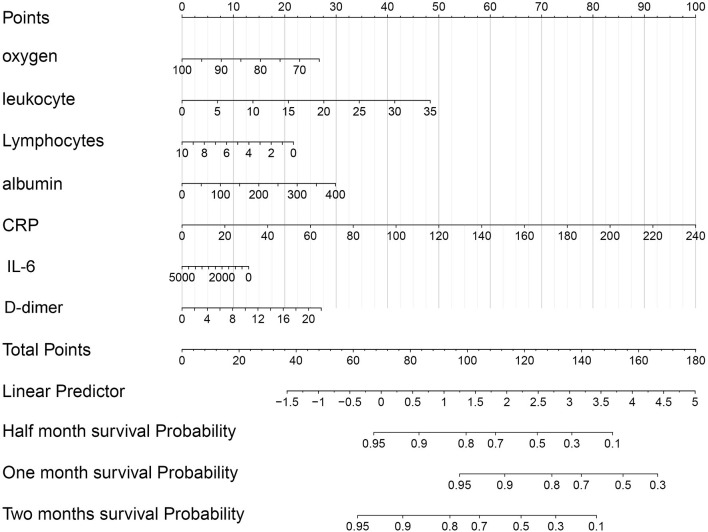
The nomogram of the seven indicators was used to predict the overall survival (OS) of COVID-19 patients.

The nomogram was established by the risk model in the training dataset (Supplementary Figure 2A). The calibration plots showed that the nomogram had a good performance in 15, 30, and 45 days (Supplementary Figures 2B–D). The decision curve analysis for nomogram showed some benefit for predicting the survival of COVID-19 patients in 15-, 30-, and 45 days, respectively (Supplementary Figures 2E–G). In addition, a nomogram based on the seven indicators was also constructed to develop a quantitative method for the prognosis of COVID-19 patients. Points were assigned to individual variables through a point scale in the nomogram. A horizontal line was used to evaluate the points of each variable and compute the scores for each COVID-19 patient by summing the points of all variables and standardizing them to a distribution from 0 to 100. The overall survival rates were then determined for COVID-19 patients in half a month, 1, and 2 months through drafting a vertical line between the total point axis and each prognosis axis, which might help clinicians to develop clinical decision-making for COVID-19 patients (Figure 8). The AUC for the nomogram in the 15-, 30-, and 45-day survival were 0.925, 0.931, and 0.946, respectively (Supplementary Figure 3).

## Discussion

The current study focused on the levels and prognosis of seven indicators including oxygen, lymphocytes, albumin, leukocyte, CRP, IL-6, and D-dimer in COVID-19 patients. Firstly, the levels of the seven indicators were compared in three different clinical types (common, severe, and critical). The findings established that the levels of lymphocytes, albumin, and oxygen saturation indicated a decreasing trend from the common to critical clinical types. The oxygen saturation level indicates the activity and quality of the lungs. Therefore, high oxygen saturation levels are indicative of healthy lungs (17, 18). This, therefore, explains why the oxygen saturation levels were decreased from the common to critical clinical types. Moreover, the levels of lymphocytes represent the immune infiltration levels, an indication that critical clinical type patients may have suffered from immunity disorders. Therefore, the levels of lymphocytes decreased from the common to critical clinical type patients. Similarly, the albumin levels exhibited a downward trend from the common to critical clinical types (19, 20). However, the findings of the current study established that the levels of leukocytes, CRP, IL-6, and D-dimer had a reverse trend, where they significantly increased from the common to critical clinical type cases. These indicators are mainly inflammatory factors. Therefore, severe COVID-19 patients have higher indicator levels (21–23). In addition, the current study evaluated the diagnostic power of the seven indicators between the critical type and non-critical clinical types. Notably, all indicators showed a high diagnostic ability, especially CRP (AUC = 0.918) and IL-6 (AUC = 0.931). These findings indicate that these indicators are potential diagnostic biomarkers of COVID-19.

To further explore the prognostic role of the indicators, univariate Cox regression and LASSO analyses were conducted to construct a two-indicator (CRP and D-dimer) prognostic signature. The patients with high-risk scores had significantly shorter survival times and were associated with more deceased cases compared with the low-risk score patients. The findings of the ROC analysis showed a high confidence AUC value, which indicated the reliability of the prognostic model. Previous studies reported the association of CRP levels with respiratory dysfunctions and death during the severe acute respiratory syndrome (SARS) outbreaks in 2002 (24). During the COVID-19 outbreak, the findings of several studies have demonstrated a positive correlation between CRP and lung lesions in COVID-19 patients, with significant CRP alterations being observed in non-survival patients (25). In addition, the most common complications including cardiac injury and acute kidney damage were directly related to the alterations of CRP levels. This may be explained by the vigorous immune response to yield numerous immune molecules and CRP. Exceeding the CRP threshold may lead to multiple organ failure in COVID-19 patients (26, 27). The findings of the current study established that the CRP levels increased from the common to critical clinical type patients, which is consistent with the earlier explanation. D-dimer is used to assess venous thromboembolism and pulmonary embolism. Elevated D-dimer levels imply the increasing risk of abnormal blood clotting (28). Previous studies reported a positive correlation between increasing D-dimer levels and COVID-19 severity (29). Patients with COVID-19 were often bedridden and presented with abnormal coagulation functions. Therefore, the vulnerability to venous thromboembolism risk, especially among the critical type patients is a possible manifestation. This explains the observation of high D-dimer levels among critical COVID-19 patients. Notably, high D-dimer levels also manifest severe viral infection. Viral infection may lead to sepsis and promote coagulation dysfunction, which is common in severe disease progression (23).

## Conclusion

The current study established that the seven indicators play important roles in COVID-19 progression and are potential diagnostic biomarkers of COVID-19. In addition, the current study developed a two-indicator prognostic signature that may improve clinical management in COVID-19 patients.

## Data Availability Statement

The original contributions presented in the study are included in the article/Supplementary Material, further inquiries can be directed to the corresponding author/s.

## Ethics Statement

The studies involving human participants were reviewed and approved by Ethics Commission of Tongji Hospital of Tongji Medical College, Huazhong University of Science and Technology. Written informed consent was not provided because written informed consent was waived for the emergency of this infectious disease.

## Author Contributions

WZho and JM designed the study. LD, WZha, FZ, CH, MY, and ZT collected the COVID-19 clinical information. LD and WZha analyzed the data. LD wrote the manuscript. YL, ZT, and WZho revised the manuscript. All authors contributed to the article and approved the submitted version.

## Funding

This research was supported by the Science and Technology Plan Project of Fujian (2020D023).

## Conflict of Interest

The authors declare that the research was conducted in the absence of any commercial or financial relationships that could be construed as a potential conflict of interest.

## Publisher's Note

All claims expressed in this article are solely those of the authors and do not necessarily represent those of their affiliated organizations, or those of the publisher, the editors and the reviewers. Any product that may be evaluated in this article, or claim that may be made by its manufacturer, is not guaranteed or endorsed by the publisher.

## Supplementary Material

Supplementary Figure 1 The evaluation of the prognosis of seven indicators using the ROC analysis.

Supplementary Figure 2 The construction and evaluation of the nomogram for predicting survival for COVID-19 patients. **(A)** The nomogram plot was established by the two prognostic indicators. The calibration plot for the internal validation of the nomogram in 15- **(B)**, 30- **(C)**, and 45 **(D)** days, respectively. The decline curve analysis (DCA) of the nomograms compared for the 15- **(E)**, 30- **(F)**, and 45 **(G)** days survival in COVID-19 patients, respectively.

Supplementary Figure 3 Evaluation of the nomogram through the ROC analysis.
