# Supplementary figures and images for: Prognostic Role and Diagnostic Power of Seven Indicators in COVID-19 Patients
Source: Front Med (Lausanne). 2021 Oct 27;8:733274. doi: 10.3389/fmed.2021.733274 (PMC8578970; doi:10.3389/fmed.2021.733274)

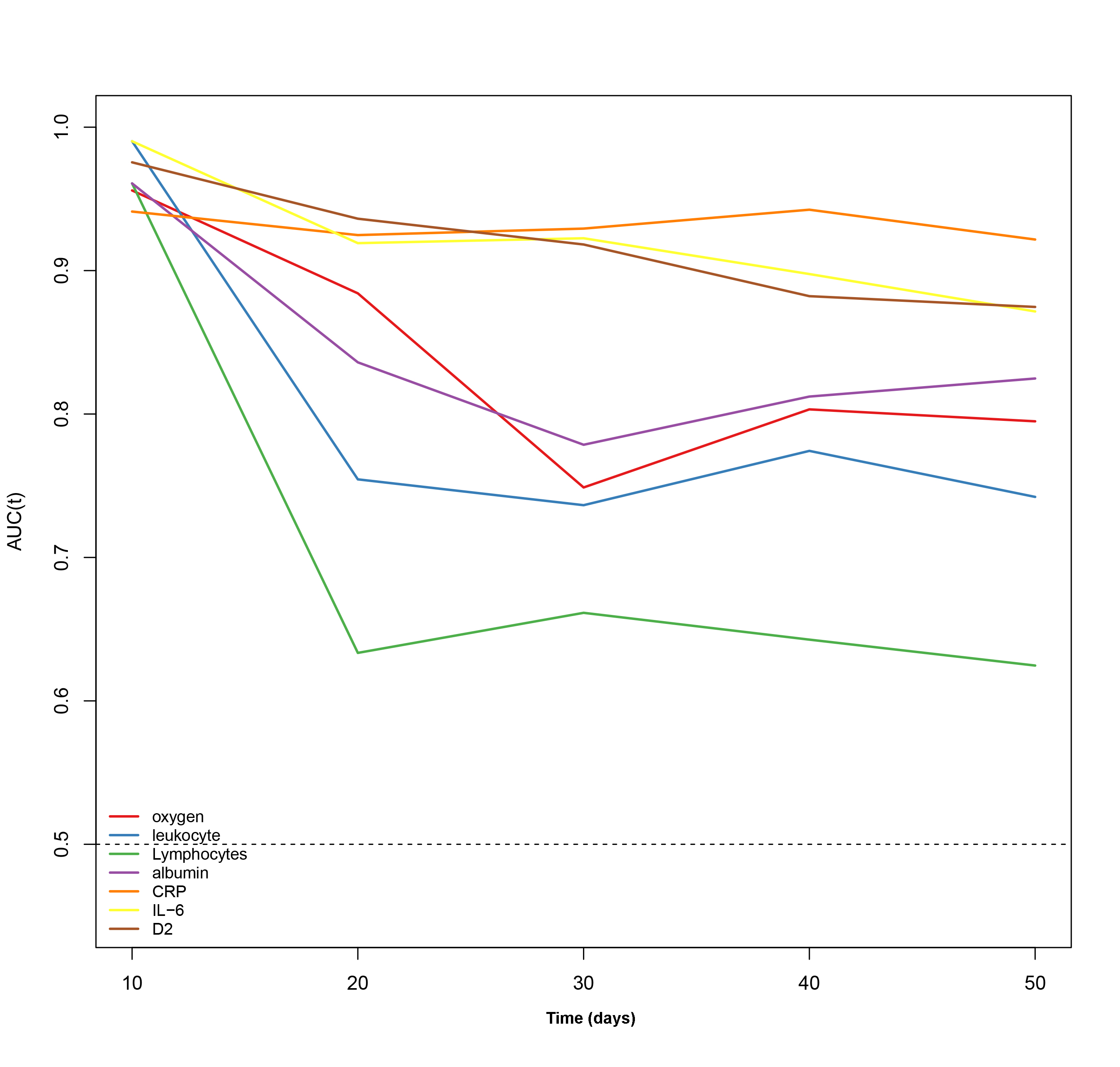

Supplement: Supplementary Figure 1 — The evaluation of the prognosis of seven indicators using the ROC analysis. [file Image_1.JPEG]

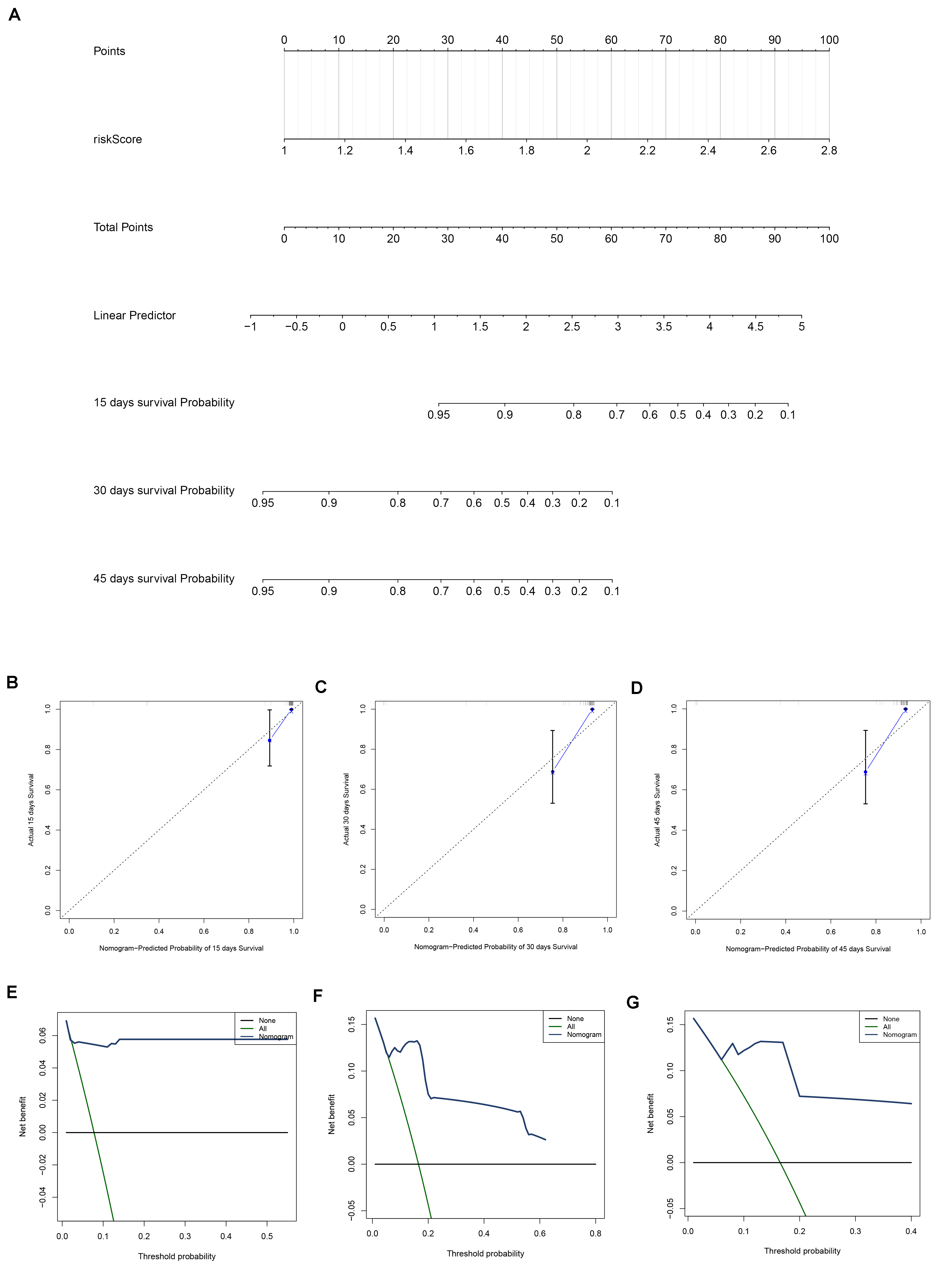

Supplement: Supplementary Figure 2 — The construction and evaluation of the nomogram for predicting survival for COVID-19 patients. (A) The nomogram plot was established by the two prognostic indicators. The calibration plot for the internal validation of the nomogram in 15- (B), 30- (C), and 45 (D) days, respectively. The decline curve analysis (DCA) of the nomograms compared for the 15- (E), 30- (F), and 45 (G) days survival in COVID-19 patients, respectively. [file Image_2.JPEG]

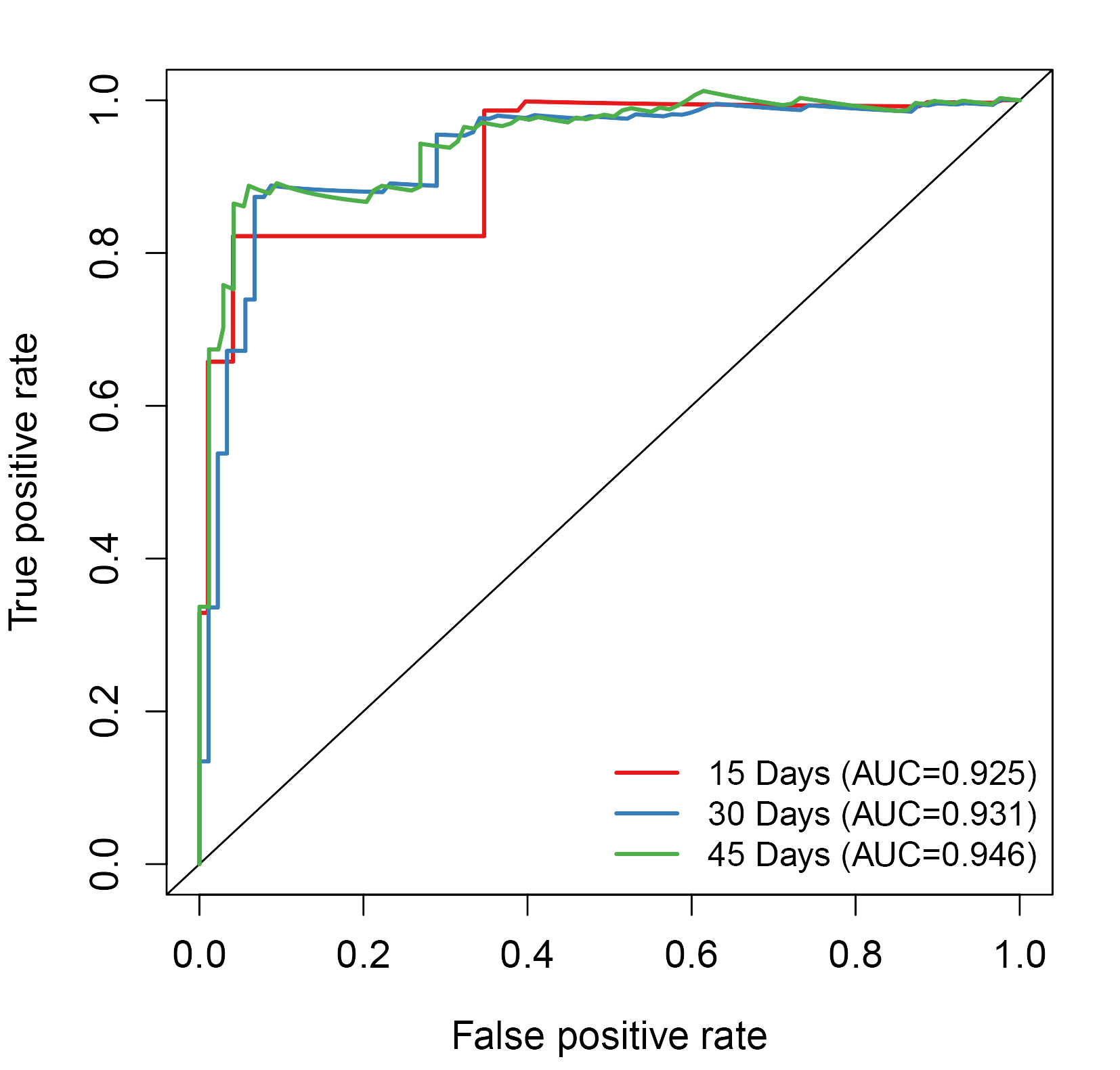

Supplement: Supplementary Figure 3 — Evaluation of the nomogram through the ROC analysis. [file Image_3.JPEG]
